# High‐Temperature Excitonic Condensation in 2D Lattice

**DOI:** 10.1002/advs.202404436

**Published:** 2024-09-06

**Authors:** Yushuo Xu, Yuanyuan Wang, Shiqiang Yu, Dongyue Sun, Ying Dai, Baibiao Huang, Wei Wei

**Affiliations:** ^1^ School of Physics State Key Laboratory of Crystal Materials Shandong University Jinan 250100 China; ^2^ Science, Mathematics and Technology Cluster Singapore University of Technology and Design Singapore 487372 Singapore

**Keywords:** 2D materials, Bose–Einstein condensation, excitonic condensation, spatially indirect exciton, superfluidity

## Abstract

Exploration of high‐temperature bosonic condensation is of significant importance for the fundamental many‐body physics and applications in nanodevices, which, however, remains a huge challenge. Here, in combination of many‐body perturbation theory and first‐principles calculations, a new‐type spatially indirect exciton can be optically generated in two‐dimensional (2D) Bi_2_S_2_Te because of its unique structure feature. In particular, the spin‐singlet spatially indirect excitons in Bi_2_S_2_Te monolayer are dipole/parity allowed and reveal befitting characteristics for excitonic condensation, such as small effective mass and satisfied dilute limitation. Based on the layered Bi_2_S_2_Te, the possibility of the high‐temperature excitonic Bose–Einstein condensation (BEC) and superfluid state in two dimensions, which goes beyond the current paradigms in both experiment and theory, are proved. It should be highlighted that record‐high phase transition temperatures of 289.7 and 72.4 K can be theoretically predicted for the excitonic BEC and superfluidity in the atomic thin Bi_2_S_2_Te, respectively. It therefore can be confirmed that Bi_2_S_2_Te featuring bound bosonic states is a fascinating 2D platform for exploring the high‐temperature excitonic condensation and applications in such as quantum computing and dissipationless nanodevices.

## Graphical abstract

A novel type of spatially indirect exciton in two‐dimensional Bi_2_S_2_Te monolayer due to its unique structure is identified. The spin‐singlet excitons are dipole/parity allowed and feature with small effective mass and large binding energy. High‐temperature excitonic Bose–Einstein condensation and superfluidity can be anticipated, offering a fascinating platform for exploring excitonic condensation and the development of excitonic dissipationless nanodevices.

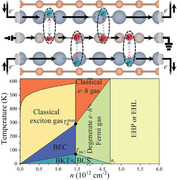

## 1 Introduction

Bose–Einstein condensation (BEC) as a macroscopic quantum coherent state exhibits exclusive features of bosonic particles, harboring appealing quantum phenomena such as superfluidity and superconductivity.^[^ ^1^, ^2^ ^]^ In addition to the exotic transport properties, researches in this field have currently focused on quantum computation,^[^ ^3^, ^4^, ^5^ ^]^ light communication, and information storage.^[^ ^6^, ^7^ ^]^ Exciton as quasiparticle (QP) refers to a bound system, in which electron and hole are combined by Coulomb interaction.^[^ ^8^, ^9^, ^10^, ^11^ ^]^ Unlike charged Cooper pairs, excitons are spatially compact and can couple directly to photons.^[^ ^12^, ^13^, ^14^, ^15^, ^16^ ^]^ Superior to the cryogenically atomic BEC in the vapor of rubidium occurring at only 170 nK,^[^ ^17^ ^]^ the characteristic small effective mass of excitons (in the order of free electron mass) implies elevated phase transition temperature of excitonic condensation.^[^ ^18^, ^19^ ^]^ In comparison to the atomic BEC, as a consequence, excitonic BEC with the bosonic excitons condensed into the lowest‐energy state has been raising increased interest.^[^ ^20^, ^21^, ^22^ ^]^ Unraveling the formation and corresponding mechanism of excitonic condensation signifies great significance in many‐body physics and indicates great potential in applications.

**Figure 1:**
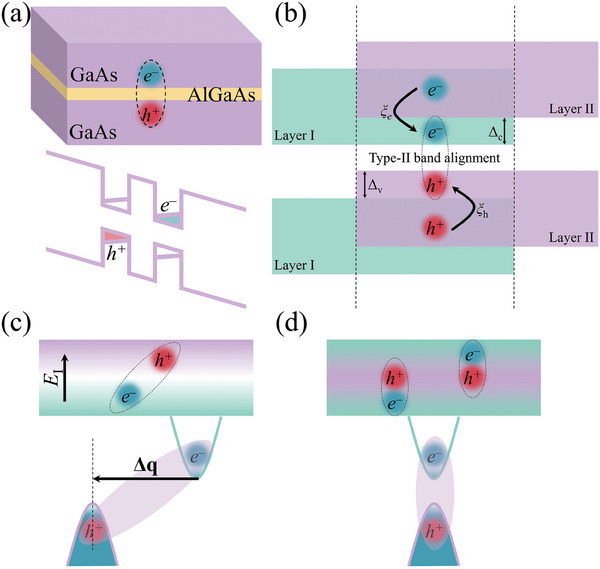
a) Schematic of the formation of indirect exciton in QWs through electron/hole doping. b) Illustration of the type‐II band alignment and spatially indirect exciton for vdW bilayers. Band offsets and ultrafast charge transfer are denoted. c) Finite‐momentum indirect exciton in the polarized system with an indirect band gap. d) Sketch of the spatially indirect exciton in multi‐atomic‐layer 2D materials, in which the band edges are contributed by separated atomic layers, and the excitons are formed by direct vertical transitions.

In previous works, the creation and manipulation of the macroscopic quantum condensation of indirect excitons were experimentally conducted based on the coupled quantum wells (CQWs). In particular, doped electrons and holes are confined in separated GaAs quantum wells with a thin AlGaAs barrier.^[^ ^23^, ^24^ ^]^ In CQWs, nevertheless, the electron‐hole interaction is comparatively weak due to the large distance in order of dozens of angstroms,^[^ ^25^ ^]^ see **Figure** **1**a. In addition, the created indirect excitons can capture one extra electron (hole) with high probability, giving rise to negative (positive) trions and placing obstacles for the observation of the excitonic BEC.^[^ ^26^ ^]^ Furthermore, for the CQWs the rough interface also has a striking influence on the exciton behavior.

To circumvent these problems, the electron‐hole pairs should be bound strongly by Coulomb attraction to suppress the dissociation, and, simultaneously, the wave functions of electron and hole ought to have a small overlap to reduce the exciton annihilation (long exciton lifetime). In this view, on one hand, van der Waals (vdW) homo‐ and hetero‐structures of two‐dimensional (2D) materials (i.e., transition metal dichalcogenides) have been experimentally investigated.^[^ ^27^, ^28^, ^29^ ^]^ In the vdW architectures, spatially indirect excitons with tight binding and small overlap can be generated because of the weak dielectric screening and the spatial separation of electron and hole. In a heterobilayer composed of 2D MoSe_2_ and WSe_2_, for instance, the critical temperature of excitonic BEC is speculated to be at least two orders of magnitude higher than that of free elementary bosons, achieving 3.5–100 K.^[^ ^30^ ^]^

Although the designed vdW homo‐ and hetero‐structures constituted by monolayer materials possess sharp interfaces and can shorten the separation distance, the optical formation of the indirect excitons generally undergoes a two‐step process rather than a direct transition. As shown in Figure 1b, intralayer excitons are generated first, and then the hot carriers relax to opposite layers across the vdW gap, finally creating the so‐called charge‐transfer excitons. In this case, the interlayer photoexcitation probability is two orders of magnitude smaller than that of intralayer excitation. In addition, transition metals primarily contributing to the band edges are surrounded by chalcogens, and, therefore, the spatial hindrance further aggravates the charge‐transfer process.^[^ ^31^ ^]^

The charged trions in CQWs and the low formation efficiency of indirect excitons in vdW structures fatally resist the excitonic condensation. Even though efforts have been made both in theory and experiments based on CQWs and vdW systems, a new strategy for the generation of spatially indirect excitons is fundamentally important for high‐temperature excitonic physics, especially in two dimensions. In consideration of the requirement of the formation of spatially indirect excitons, on the other hand, polarized III_2_‐VI_3_ monolayers (such as In_2_Se_3_) could probably be potential candidates. In these materials, the out‐of‐plane electric polarization can separate electron and hole into different atomic layers, giving rise to the formation of spatially indirect excitons. However, these materials are usually featured by indirect band gap,^[^ ^32^, ^33^ ^]^ and, therefore, the momentum mismatch seriously impairs the transition probability (Figure 1c). In addition, a recent pioneering work on excitonic insulator has demonstrated that the detection of the condensation of excitons with finite momentum is usually interfered by the formation charge density wave (CDW).^[^ ^19^ ^]^ It has been theoretically concluded that, furthermore, the periodic lattice distortion poses an obstacle for the characterization of exciton condensation by introducing a CDW gap, and a CDW state can greatly affect the excitonic characteristics.^[^ ^34^, ^35^ ^]^ It is of interest that, therefore, the calculation approaches and/or general models used in these works can provide alternative ways for the further study of excitonic condensation.

In this work, we go beyond the conventional paradigm and find a new way for the formation of spatially indirect excitons in single‐phase monolayer structures, rather than the artificial CQWs and vdW structures (Figure 1d). We screened the bismuth‐based Bi_2_X_2_Y monolayers (X ≠ Y = S, Se, and Te) due to their quintuple atomic‐layer structure and layer‐resolved band edge contribution, and we found that spatially indirect excitons and high‐temperature excitonic condensation can be presented in Bi_2_S_2_Te monolayer. The atomic‐layer separation of the excited electrons and holes endows the indirect exciton not only small wave function overlap but also relatively strong Coulomb interaction. In particular, the small exciton effective mass originating from the unique band edge dispersion and the dipole/parity allowed strong transition guaranteed by the inversion symmetry mark Bi_2_S_2_Te as a preferable platform to investigate the excitonic condensation in two dimensions. Importantly, the exciton dilute limitation is satisfied in this material in two dimensions, and the exciton lifetime is very encouraging for the discussion of excitonic condensation. It is of high interest that, for 2D Bi_2_S_2_Te, the phase transition temperatures for excitonic BEC and superfluidity are predicted to be 289.7 and 72.4 K, respectively, which are dramatically higher than those previously reported. It therefore can be confirmed conclusively that our work opens up a new direction for the high‐temperature excitonic condensation and paves the way for novel applications in such as dissipationless optoelectronic nanodevices.

## 2 Results and Discussion

**Figure 2:**
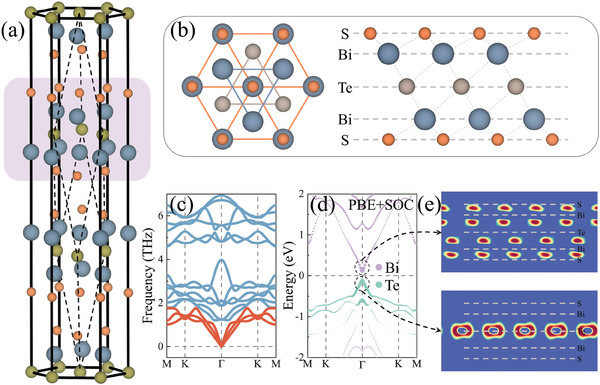
a) Layered bulk structure of Bi_2_X_2_Y (X ≠ Y = S, Se, and Te) with the blue‐gray, orange, and dark‐green spheres representing Bi, X, and Y atoms, respectively. The pink region denotes the quintuple atomic‐layer structure of Bi_2_X_2_Y monolayer. b) Top and side views of the 2D Bi_2_S_2_Te. c) Phonon spectrum for Bi_2_S_2_Te monolayer. The branches colored in red and blue‐gray symbolize the acoustic and optical modes, respectively. d) Atomic‐resolved band structure considering SOC for Bi_2_S_2_Te monolayer at PBE level of theory. e) Partial charge density for the VBM and CBM of Bi_2_S_2_Te monolayer, where the isosurface is set to a suitable value for visualizing the spatial separation.

In both experiments and theory, the ground‐state properties including the topological behaviors of Bi_2_X_2_Y have been explored.^[^ ^36^, ^37^, ^38^, ^39^, ^40^ ^]^ However, researches on the optical properties and excitonic behavior of Bi_2_X_2_Y are still missing, to the disadvantages of the fundamental understanding on the light–matter interactions of this class of materials. In **Figure** **2**a, layered bulk structure of Bi_2_X_2_Y with a space group of $R\bar{3}m$ (No.166) is shown. It can be found that layered Bi_2_X_2_Y is composed of quintuple X–Bi–Y–Bi–X monolayers. In Figures S1–S4 (Supporting Information), band structures at PBE level of theory for bulk and 2D forms Bi_2_X_2_Y with and without the consideration of spin‐orbit coupling (SOC) are presented. It can be seen that most of the Bi_2_X_2_Y monolayers exhibit indirect band gaps with the conduction band minimum (CBM) located at Γ point and the valence band maximum (VBM) positioned at a *k* point along the high‐symmetry line Γ–K. In case we take SOC into account, band structures change dramatically. Especially, an indirect‐direct bandgap transition occurs for the Bi_2_Se_2_Te monolayer. This can be rationalized by the different atomic contributions to band edges as well as the distinct SOC strength, which accounts for the band dispersion variation. It is of interest to see that Bi_2_S_2_Te in both bulk phase and 2D limitation reveals a direct band gap, and the slight difference in band dispersion around the Fermi level implies layer‐insensitive electronic properties. This can be explained by the interlayer weak vdW interactions. In addition, atoms contributing to the band edge states (see below) are encapsulated by atomic S layers, and thus the band structure is largely resistant to the change of layer number. Generally, a direct band gap can be optically excited without an auxiliary phonon, which, therefore, is preferable for the high‐temperature excitonic condensation. In consideration of the above discussion, we take Bi_2_S_2_Te monolayer as a representative to unravel the formation mechanism of the spatially indirect excitons and the high‐temperature excitonic condensation.

In Figure 2b, the structure of Bi_2_S_2_Te monolayer is shown. It can be found that the primitive cell of the monolayer limitation consists of five atomic layers in a sequence of S–Bi–Te–Bi–S, belonging to the space group of $P\bar{3}m1$ with *D*_3d_ point symmetry. The atomic Te layer is sandwiched by two BiS layers, and there are two inversion centers located at Te position and the midpoint of two Te atoms. To mimic the experimentally mechanical exfoliation of Bi_2_S_2_Te monolayer from its layered bulk phase, exfoliation energy is calculated and the results are illustrated in Figure S5 (Supporting Information). The exfoliation energy for Bi_2_S_2_Te is certified to be 9.6 meV Å^−2^, which is comparable to that of graphene (12 meV Å^−2^) and fairly smaller than that of MoS_2_ (26 meV Å^−2^). This is a firm manifestation of high feasibility for experimental exfoliation of Bi_2_S_2_Te monolayer from its layered bulk counterpart. In addition, the phonon dispersion calculation and ab initio molecular dynamics simulation are conducted for Bi_2_S_2_Te monolayer to confirm its dynamic and thermal stability, respectively. As shown in Figure 2c, the phonon spectrum shows no imaginary modes for both the acoustic and optical branches over the entire Brillouin zone, verifying the dynamic stability. Figure S6 (Supporting Information) indicates that the material exhibits neither structural distortion nor bond breaking under 300 K after 5 ps, thus implying the thermal stability.

In centrosymmetric monolayers, the parity of band edges, denoted as even (eigenvalue, +1) or odd (eigenvalue, –1) for the wave function under inversion operation, plays a crucial role in the optical response of the materials. In case the wave functions of the valence and conduction bands have the same parity, a negligible optical dipole transition will take place on account of the symmetry constraint.^[^ ^41^ ^]^ Under a perturbing electromagnetic field, interband transition matrix follows ***M***_vc**q**_ (***k***) = 〈*c*, ***k*** + ***q*** | *e* ^i**q** · **r**^∇|*v*, ***k*** 〉, which can be generally simplified as transition dipole moment $\langle \varphi_{h}\left|\hat{\mu }\right|\varphi_{e}\rangle$ with the electric dipole approximation (Supporting Information). It demonstrates a transition from the initial state |φ_e_〉 to the final state |φ_h_〉, with $\hat{\mu }$ being the electric dipole operator (Supporting Information). In this sense, the dipole moment of the band edge transition should be nonzero so that the excitons can be optically excited. Therefore, the parities of the initial and final states are supposed to be opposite to have a finite transition dipole moment because $P^{†}\hat{\mu }P=-\hat{\mu }$, where P represents the parity operator.

**Figure 3:**
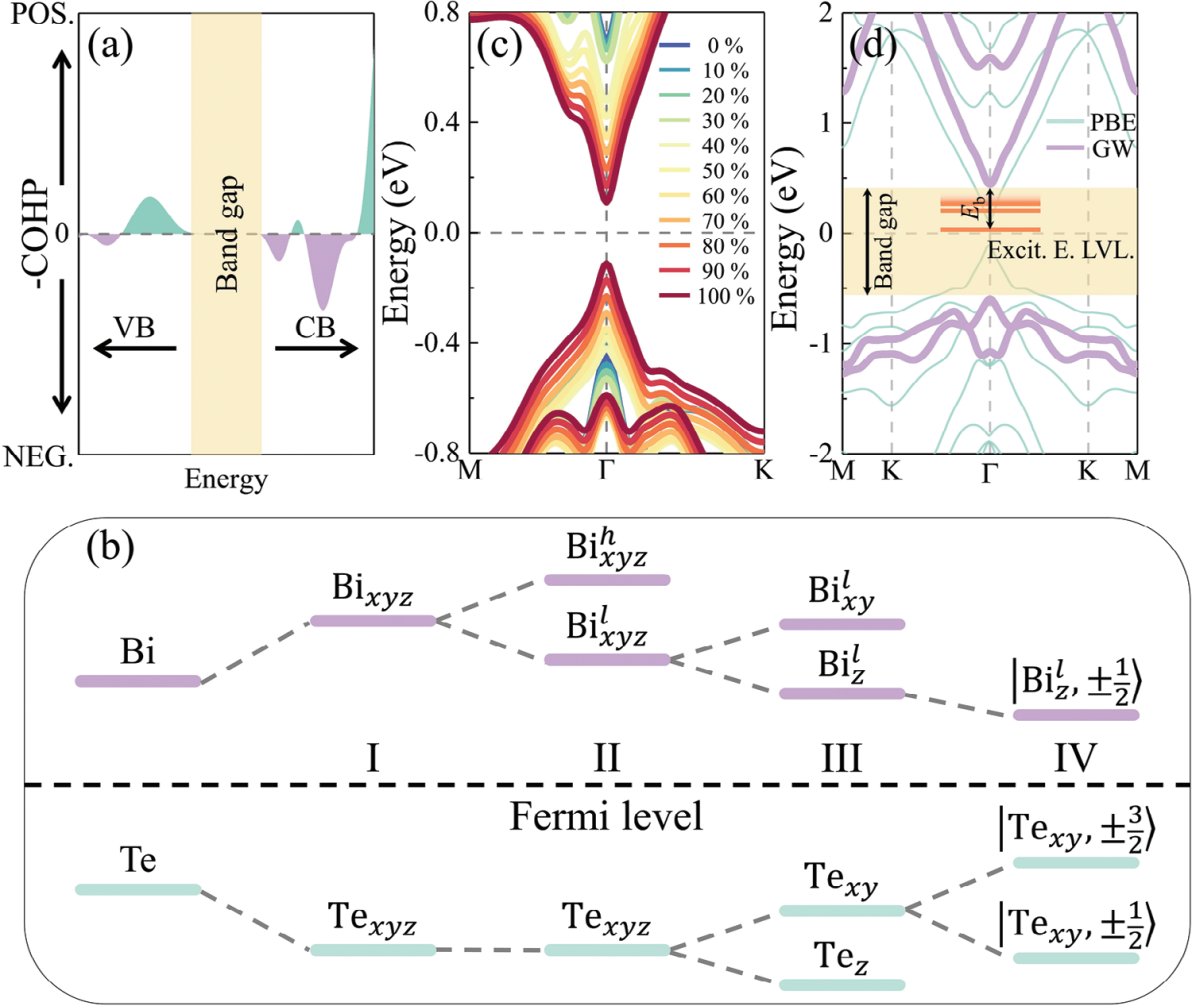
a) Crystal orbital Hamilton population with positive and negative values being the bonding and antibonding states, respectively. b) Schematic of orbital energy diagram. Stage I represents an energetic repulsion between Bi and Te atom. Stage II shows the orbital energy splitting due to the interaction between Bi atoms, and the orbital energy remains degenerate for Te atom. Stage III considers the crystal field effect. Stage IV illustrates the effect of SOC. c) Evolution of band dispersion with different SOC strength. d) Band structure of Bi_2_S_2_Te monolayer at GW level of theory, and that from PBE is also shown for comparison.

In Figure 2, the fat band structure and partial charge densities for the VBM and CBM of Bi_2_S_2_Te monolayer are presented. It clearly shows that the charge density of VBM and CBM distribute primarily on the atomic Te and Bi layer, respectively, indicative of substantial separation between electron and hole. In sharp contrast to the direct excitons (e.g., intralayer excitons), a permanent dipole moment is assigned to the spatially indirect exciton.^[^ ^42^, ^43^ ^]^ It can be seen that VBM is primarily contributed by the *p*_x_ and *p*_y_ orbitals of Te atom, while CBM is dominated by the *p_z_* states of Bi atom. It is well convinced that the wave functions of *p_i_* (*i*  =  *x*, *y*, *x*) orbitals satisfy $P\;|p_{i}\rangle =\left|-p_{i}\rangle =-\right|p_{i}\rangle$; that is, the odd parity. Consequently, it appears that the parity symmetries of the band edges dictate the lowest‐energy optical transition to be dipole‐forbidden. In accordance to our calculation results; however, Bi_2_S_2_Te monolayer reveals a sizable excitonic absorption on the low energy side. We rationalize this contradiction by molecular orbital theory. It should be noted that, for Bi_2_S_2_Te monolayer, Bi atoms contributing to the CBM are from the upper and lower Bi layers; while they are nonequivalent. It can be observed straightforwardly from the crystal orbital Hamilton population that the molecular orbsitals constituting the CBM manifest antibonding features (see **Figure** **3**a). In this view, the electronic wave function can be written as

(1) $$\varphi^{\mathrm{CBM}}=\frac{1}{\sqrt{2}}\;\left(\left|Bi^{u},p_{z}\rangle \right.-\left|Bi^{l},p_{z}\rangle \right.\right)$$

where Bi^*u*^ and Bi^*l*^ stand for the Bi atoms from upper and lower Bi layers, respectively. Under inversion operation, we will have

(2) $$P\;\varphi^{\mathrm{CBM}}=\varphi^{\mathrm{CBM}}$$

where $P\;|Bi^{u},p_{z}\rangle =-|Bi^{l},p_{z}\rangle$ and $P\;|Bi^{l},p_{z}\rangle =-|Bi^{u},p_{z}\rangle$. Therefore, the wave function of the CBM characters actually the even parity, and the lowest‐energy optical transition in Bi_2_S_2_Te monolayer is dipole‐allowed. This lays the foundation for the formation of spatially indirect excitons in Bi_2_S_2_Te monolayer. In Figure 3b, an orbital diagram is provided for the Bi_2_S_2_Te monolayer. It should be noted that the orbital energy of Te atom is kept degenerate on stage II, since the interaction with S atom is negligible.^[^ ^44^ ^]^

The SOC Hamiltonian can be expressed as *H*_SO_ =  λ ***S*** · ***L***, considering only the on‐site coupling. It can be known from the band structure of Bi_2_S_2_Te monolayer that SOC causes the dramatic variations in band dispersion and energy gap. To elucidate how SOC can drastically modify the band structure, band dispersion evolution as a function of the SOC strength is attained, as shown in Figure 3c. It can be seen that the band gap gradually decreases as the SOC strength increases. It is therefore the strong SOC of Te atom that accounts for the unique band structure of Bi_2_S_2_Te monolayer. The significantly small carrier effective mass, which is of paramount importance for the discussion of excitonic condensation, can also be attributed to the strong SOC of Te atom.

In order to obtain the correct excited‐state properties such as the optical absorption spectrum of Bi_2_S_2_Te monolayer, we consider the GW approximation based on the many‐body perturbation theory. The general nonlinear QP equation reads

(3) $$E_{nk}^{\mathrm{QP}}=\varepsilon_{nk}+\langle \psi_{nk}|\Sigma \left(E_{nk}^{\mathrm{QP}}\right)-V_{\mathrm{xc}}|\psi_{nk}\rangle$$

where ε_nk_ represents the Kohn–Sham eigenvalue; $\Sigma_{\mathrm{nk}}=\Sigma_{nk}^{x}+\Sigma_{nk}^{c}$, with $\Sigma_{nk}^{x}$ and $\Sigma_{nk}^{c}$ being the exchange and correlation self‐energy operators, respectively; and *V*_xc_ symbolizes the density functional theory (DFT) exchange–correlation potential.^[^ ^45^, ^46^ ^]^ In Figure 3d, band structure of Bi_2_S_2_Te monolayer at GW level of theory is presented. It can be observed that QP correction obviously renormalizes the band dispersion as well as the band gap. This is an indication that the QP correction is momentum‐dependent, rather than a simple scissor operator. In particular, the characteristic of the direct band gap of Bi_2_S_2_Te monolayer remains, indicating that CDW caused by unexpected structural distortion due to the finite momentum transfer can be significantly suppressed.^[^ ^47^, ^48^ ^]^ Specifically, PBE leads to a direct band gap of 0.22 eV at the Γ point while it turns out to be 1.05 eV after taking the electron–electron self‐energy interaction into account, corresponding to a QP correction of 0.83 eV.

**Figure 4:**
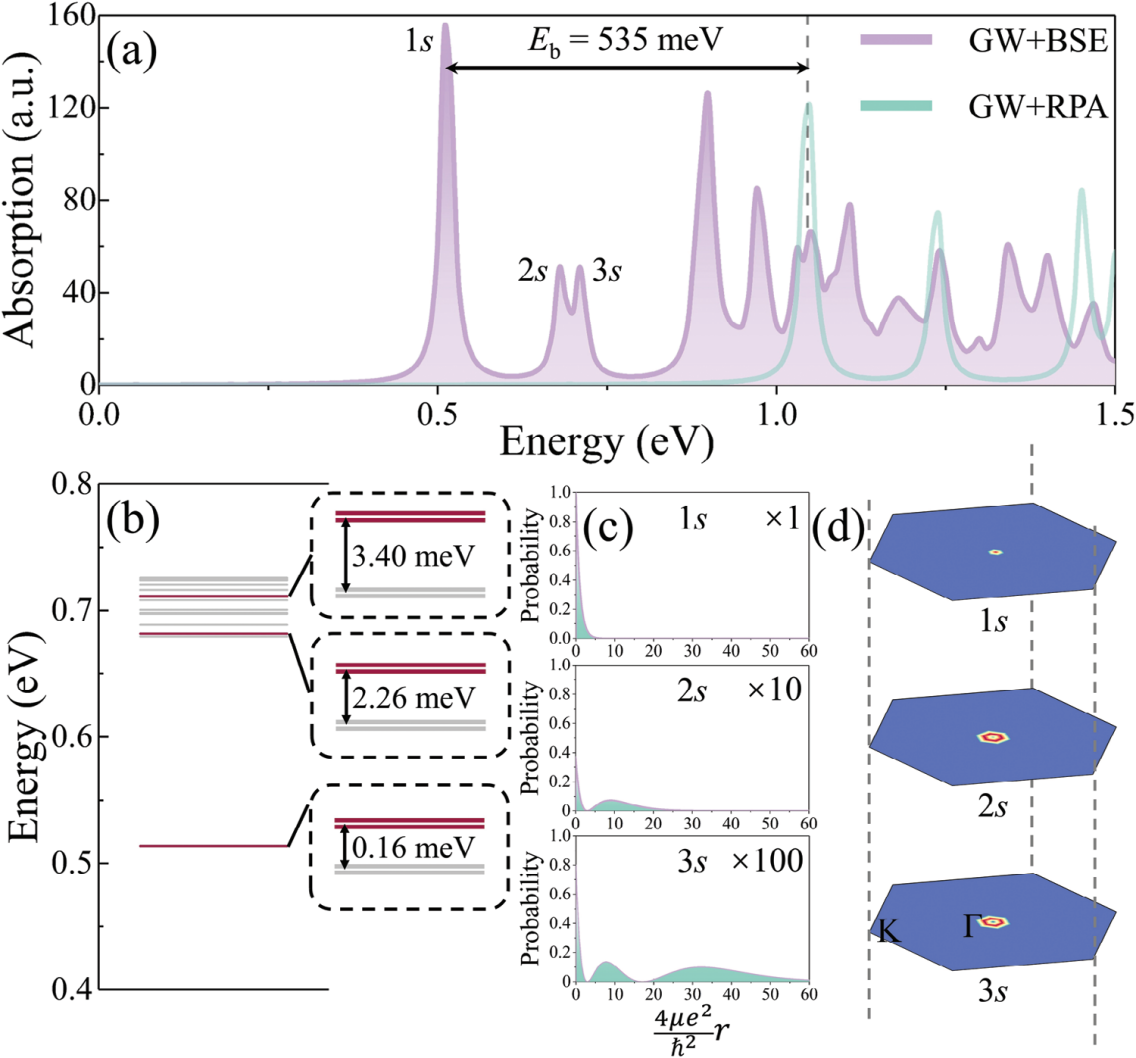
a) Optical absorption spectrum of Bi_2_S_2_Te monolayer. b) Excitation energy spectrum in a range from 0.4 to 0.8 eV, burgundy and gray lines denote the optically bright and dark excitonic absorption, respectively. c) Probability distribution function for 1*s*, 2*s*, and 3*s* excitons, with the maximum value of the 1*s* exciton set to 1. d) Distribution of 1*s*, 2*s*, and 3*s* excitons in reciprocal space.

In **Figure** **4**a, the optical absorption spectrum of Bi_2_S_2_Te monolayer is shown, which is obtained by solving the Bethe–Salpeter equation (BSE) with electron–hole interaction included on top of GW approximation (GW+BSE).^[^ ^49^, ^50^ ^]^ In order to signify the excitonic effect, optical spectrum from random phase approximation (RPA) is also provided for comparison. In case of considering the excitonic effect, the profile of the spectrum displays a large global redshift and the oscillator strength is redistributed. In particular, three typical exciton peaks appear on the low‐energy side. In line with the hydrogen‐like model, three bright excitons can be labeled as 1*s*, 2*s*, and 3*s* states with the excitation energies being 0.51, 0.68, and 0.71 eV, respectively. Regarding the first 1*s* exciton, its binding energy *E*_b_ (defined as the difference between the QP band gap and excitation energy) is 535 meV, suggesting the strongly bound nature. Such an exciton binding energy is comparable to that of transition metal dichalcogenides monolayers.^[^ ^51^, ^52^, ^53^ ^]^

In Figure 4b, the excitation energy spectrum in the range from 0.4 to 0.8 eV is accordingly illustrated, in which the first three bright excitons are colored in burgundy. The reason why only *s* states are optically bright can be explained by the selection rule proposed by Louie et al., that is, *m*  =   − *l*(mod *n*) (Supporting Information). Here, *m* is the cylindrical angular quantum number of the exciton envelope function, *l* is the winding number of interband optical transition matrix element and *n* is the discrete rotational symmetry.^[^ ^54^ ^]^ In Figure S7 (Supporting Information), the phase and amplitude of the interband optical transition matrix element are presented, which indicates a zero winding number around the Γ point. Accordingly, thus only the *s* states can be optically excited, while the *p* and *d* exciton states formed by the vertical transitions near Γ point are optically dark. The excitation energies (0.51, 0.68, and 0.71 eV for 1*s*, 2*s*, and 3*s* states, respectively) essentially follow the 2D hydrogen‐like stationary state energy, and the deviation originates mainly from the distinct dielectric screening for different excitons (Supporting Information). In accordance to the 2D hydrogen‐like atom model, we plot the probability distribution functions for the 1*s*, 2*s*, and 3*s* excitons. It can be seen that the excitons show damping features, and the radius increases from 1*s* to 3*s* states, see Figure 4c. In particular, four bound excitons constitute the nearly degenerate 1*s* state, which can be categorized into doubly degenerate dark excitons (spin‐triplet exciton) and doubly degenerate bright excitons (spin‐singlet exciton). It can be known that the dark excitons are respectively 0.16, 2.26, and 3.40 meV lower in energy than the corresponding bright ones, which can be attributed to the electron–hole exchange interaction. In addition, the increase in singlet–triplet energy difference as the principle quantum number becomes larger generally originates from the wave function overlap between electron and hole, as well as the distinct dielectric screening by virtue of different exciton radius. It should be noted that the double‐degenerate bright excitons can be transformed to energetically preferable dark excitons through electron–electron (hole–hole) exchange interaction.^[^ ^55^, ^56^ ^]^ Similar results can also be attained for the 2*s* and 3*s* states.

**Figure 5:**
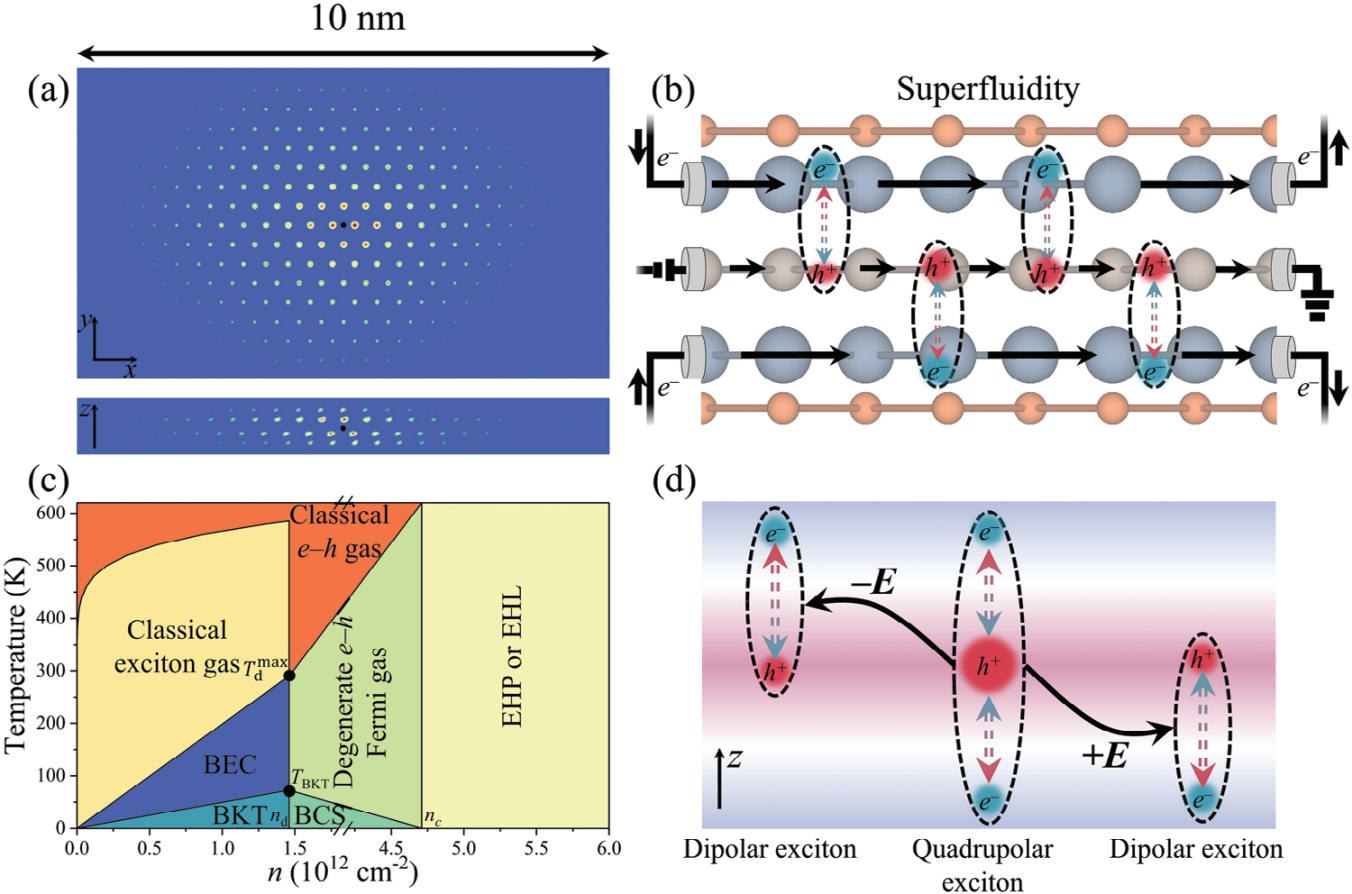
a) Top and side views of the distribution of the 1*s* exciton in real space. b) Schematic of the excitonic counter‐flow current effect in Bi_2_S_2_Te monolayer. c) Phase diagram for 2D Bi_2_S_2_Te under different exciton density and temperature. d) Electric control of the quadrupolar exciton in Bi_2_S_2_Te monolayer.

In Figure 4d, the exciton envelope functions for the 1*s*, 2*s*, and 3*s* states in reciprocal space are provided, which elucidate that the optical transition occurs solely at the Γ point. It is also indicative of a relatively large exciton radius in real space. In order to visualize the bound feature of the lowest‐energy 1*s* exciton, exciton wave function in real space is acquired by diagonalizing the BS two‐particle Hamiltonian

(4) $$\;{\left|\left|\psi^{\lambda }\left(r_{e},\;r_{h}\right)\right.\right|}^{2}={\left|\sum_{\mathrm{vc}k}A_{\mathrm{vc}k}^{\lambda }\varphi_{ck}\left(r_{e}\right)\varphi_{v-k}^{*}\left(r_{h}\right)\right|}^{2}$$

where λ, *e*, *h*, *v*, *c*, *A*, and φ represent the exciton index, electron, hole, valence band, conduction band, exciton amplitude and electronic wave function, respectively. In **Figure** **5**a, the spatial distribution of the 1*s* exciton is shown. It can be seen that, when the quasi‐hole is fixed at the middle Te layer, quasi‐electrons are distributed on the two outside Bi layers and exhibit a damping landscape. This immediately confirms the formation of spatially indirect excitons in Bi_2_S_2_Te monolayer, benefiting the small wave function overlap and long lifetime. The root mean square radius *r*_RMS_ of the 1*s* exciton is estimated to be 2.6 nm, which, as shown below, is sufficiently large for the discussion of the excitonic condensation.

It has been empirically clarified that the dissociation temperature of an exciton is in a close relation with its binding energy, that is, *k*_B_ *T*_d_  ≈ 0.1*E*_b_ with *k*_B_ being the Boltzmann constant.^[^ ^57^ ^]^ It should be noted that the discrepancy is due primarily to the ground‐state nature of the used approaches. In case a dynamical process is considered, for example, the electron–phonon coupling, results will differ in absorption strength and excitation energy. In particular, absorption strength varies with temperature and the excitation energy will be slightly blue/red shifted, which have been extensively theoretically explored.^[^ ^58^, ^59^, ^60^ ^]^ It has been confirmed that, however, the influence of electron–phonon coupling on the zero‐momentum direct optical transitions is relatively weak. In addition, time‐dependent DFT and nonadiabatic molecular dynamics are also commonly used to study the excitonic dynamics, e.g., the transfer and recombination of photoexcited carriers.^[^ ^61^, ^62^, ^63^, ^64^ ^]^ In the present work, we emphasize that the excitonic condensation behaviors obtained from the ground‐state approaches without considering dynamics processes are considerably reliable, as clarified in previous works.^[^ ^65^, ^66^, ^67^ ^]^ In this view, therefore, large binding energy suggests that the exciton can survive at high temperatures. In regard to Bi_2_S_2_Te monolayer, such a dissociation temperature for the lowest‐energy 1*s* exciton is correspondingly 620 K, significantly higher than the room temperature. To preserve the composite QP characteristics to achieve excitonic condensation, the exciton density should be smaller than a critical value

(5) $$n_{c}\;\approx \frac{1}{\pi r_{\mathrm{RMS}}^{2}}$$

For Bi_2_S_2_Te monolayer, the critical density is ≈4.71 × 10^12^ cm^−2^. When excitons condense to a degenerate state, they should maintain dilute to guarantee the bosonic nature; this is an essential prerequisite to realize the excitonic condensation. The dilute condition can be expressed as^[^ ^68^ ^]^

(6) $$\ln\ln\left(1/n_{d}r_{\mathrm{ex}}^{2}\right)\gg 1$$

where *r*_ex_ denotes the exciton radius and satisfies (Supporting Information)

(7) $$\;\frac{r_{\mathrm{RMS}}}{r_{\mathrm{ex}}}=\frac{\sqrt{3/8}}{1/2}\approx 1.225$$

In the case for the 1*s* exciton of Bi_2_S_2_Te monolayer, accordingly, *n*_d_ is estimated to be 1.46 × 10^12^ cm^−2^.

Apart from exciton density, the temperature also plays a crucial role in determining whether the excitonic BEC can be realized or not. Under an approximation of ideal 2D Bose gas, the characteristic temperature is correlated with the exciton effective mass *M*_ex_ and we have^[^ ^69^ ^]^

(8) $$k_{B}\;T_{d}^{\max}=\frac{2\pi \hbar^{2}}{M_{\mathrm{ex}}}\;n_{d}$$

where *M*_ex_ = *m*_e_  + *m*_h_. In fact, the cone‐like band dispersion of Bi_2_S_2_Te monolayer has already intuitively indicated the rather small effective masses for both electrons and holes around the Γ point. Including QP corrections, the effective masses are calculated to be *m*_e_ =  0.12 *m*_0_ for quasi‐electron and *m*_h_ =  0.16 *m*_0_ for quasi‐hole, here *m*_0_ symbolizes the free electron mass. In addition to the first‐principles calculations, the effective masses of exciton, electron and hole can also be obtained within the widely used effective mass approximation.^[^ ^70^, ^71^, ^72^, ^73^ ^]^ It should be noted that the effective masses especially derived from the QP band dispersion are substantially reliable and match well with experimental observations, which also adhere to the 2D hydrogen atom model (Supporting Information).^[^ ^10^, ^74^ ^]^ The exciton effective mass of Bi_2_S_2_Te monolayer is considerably smaller than that of the characteristic A/B exciton (0.499/0.545 *m*_0_) in MoS_2_ monolayer;^[^ ^75^ ^]^ thereby, suggesting high phase transition temperature. In the present work, we highlight that a record‐high theoretical characteristic temperature of 289.7 K can be attained for the Bi_2_S_2_Te monolayer, which demonstrates that a phase transition from a trivial state to the excitonic BEC can occur nearly at room temperature.

Within the excitonic BEC region, excitons can also enter into a superfluid state, where exciton current moves without friction at a low exciton density, namely, the Berezinskii–Kosterlitz–Thouless (BKT) phase. The phase transition temperature as a function of exciton density can be expressed as^[^ ^76^ ^]^

(9) $$k_{B}\;T_{\mathrm{BKT}}=\frac{\pi \hbar^{2}}{2M_{\mathrm{ex}}}\;n_{d}$$

In respect to Bi_2_S_2_Te monolayer, accordingly, the upper‐limit temperature permitting the superfluidity at the dilute condition is 72.4 K. We emphasize that the high critical temperatures render Bi_2_S_2_Te monolayer to be a promising platform superior to other materials for the investigations on high‐temperature excitonic condensation.^[^ ^21^, ^22^ ^]^ In a superfluid state, quasi‐electron current and quasi‐hole current can be detected due to the quintuple atomic‐layer structure of Bi_2_S_2_Te monolayer. As the atomic sublayers of Bi_2_S_2_Te is contacted with electrode, the exciton superfluidity can be tuned artificially by an external voltage gate. This is referred to as the counter‐flow current effect.^[^ ^77^ ^]^ In Figure 5b, such a device is schematically shown.

In case the density increases, the composite bosonic feature of the excitons will be gradually degraded, and, consequently, a transition from strong coupling BKT phase to weak coupling Bardeen–Cooper–Schrieffer (BCS) phase occurs.^[^ ^78^, ^79^ ^]^ When the exciton density increases further, enhanced dielectric screening will trigger a phase transition to the electron–hole liquid (EHL) or electron–hole plasma (EHP), instead of excitonic condensation. It should be emphasized that the small exciton effective mass and dipole repulsion will lead to a low possibility for the formation of biexcitons or trions, and the occurrence of EHL or EHP phase in high‐density region could also be largely prevented. In Bi_2_S_2_Te monolayer, therefore, the stable BEC/BKT phase will be highly pronounced. In other words, the bosonic excitons will condensate into the lowest‐energy state, where excitons are in a macroscope quantum coherent state rather than a series of discrete QPs.

In Figure 5c, accordingly, a phase diagram with respect to the exciton density and temperature is drawn for the Bi_2_S_2_Te monolayer. It clarifies clearly that the material displays diverse QP behaviors. Additionally, exciton of low density will dissociate above the Saha temperature, which can be generally described by the following expression^[^ ^80^ ^]^

(10) $$k_{B}T_{s}∼\left(\frac{\pi \hbar^{2}}{M_{\mathrm{ex}}}n_{\mathrm{ex}}\right)e^{\frac{E_{b}}{k_{B}T_{s}}}$$

here *n*_ex_ represents the exciton density (Supporting Information).

In order to detect and manipulate the indirect excitons experimentally, long exciton lifetime is a key precondition. In accordance to the Fermi's Golden rule, the radiative decay rate γ_S_(***Q***) for S exciton state with a wave vector of ***Q*** can be acquired.^[^ ^81^, ^82^, ^83^ ^]^ In case we take only the direct transition into account, i.e., ***Q *** =  0, the exciton lifetime is

(11) $$\tau_{S}\;\left(0\right)=\gamma_{S}^{-1}\left(0\right)=\frac{\hbar^{2}cA_{\mathrm{uc}}}{8\pi e^{2}E_{S}\left(0\right)\mu_{S}^{2}}$$

where ℏ, *c*, *A*_uc_, *E*_S_(0) and µ_S_ symbolize the reduced Planck constant, light speed, area of the unit cell, excitation energy and the modulus of the exciton transition dipole divided by the number of *k* points, respectively. In regard to the 1*s* exciton of Bi_2_S_2_Te monolayer, the lifetime is 38.4 ns at zero temperature. In general, increasing the temperature will benefit the average lifetime of excitons, following the expression^[^ ^82^, ^83^ ^]^

(12) $$\tau_{S}\;\left(T\right)=\tau_{S}\;\left(0\right)\cdot \frac{3}{4}\left(\frac{2M_{\mathrm{ex}}c^{2}}{E_{S}^{2}\left(0\right)}\right)k_{B}T$$

Accordingly, the exciton lifetime at critical temperature (289.7 K) for the BEC of Bi_2_S_2_Te monolayer turns out to be 781.0 µs, which is long enough for experimental observation. The preferable exciton lifetime makes Bi_2_S_2_Te monolayer more promising for the investigation of high‐temperature excitonic condensation.

In respect to the Bi_2_S_2_Te monolayer, the spatially indirect excitons can be regarded as quadrupolar excitons.^[^ ^84^, ^85^, ^86^ ^]^ In recent studies, quadrupolar exciton was defined and identified in vdW heterotrilayers based on transition metal dichalcogenides.^[^ ^84^, ^85^, ^86^, ^87^, ^88^ ^]^ In comparison to the vdW architectures, Bi_2_S_2_Te is the first case reported for quadrupolar excitons residing in a single‐phase monolayer structure, providing a new, promising platform for further study of quadrupolar excitons. It is highly appealing that, for instance, the complexity, low yield and undesirable by‐products related to the trilayer fabrication can be largely circumvented. In addition, the monolayer structure can also eliminate the drawbacks owing to the interface contamination, rotation angle and stacking mode.^[^ ^87^, ^88^, ^89^ ^]^ It is of interest that quadrupolar excitons can transform to dipolar excitons under an external electric field, as shown in In Figure 5d, thus deserves further study. In particular, the moment direction of the dipolar excitons can be switched by reversing the electric field, realizing probably the photo‐induced electric polarization switching. In addition, Bi_2_Se_2_Te monolayer shares the similar ground‐state properties with Bi_2_S_2_Te monolayer, and the significant SOC of Te atom forces a transformation from Mexican hat‐like valence band to a cone‐like dispersion around the Γ point. This corresponds to a small exciton effective mass, which is a strong indication that Bi_2_Se_2_Te monolayer is also an alluring candidate for the high‐temperature exciton condensation in two dimensions.

## 3 Conclusion

In summary, Bi_2_S_2_Te monolayer is proven to be a preferable material for the investigation of high‐temperature excitonic condensation. In the light of SOC effect and parity analysis, the lowest‐energy spatially indirect exciton is spin/parity/dipole allowed and can directly couple to a photon. Because of the strong SOC and electron–electron self‐energy interaction, the lowest‐energy spatially indirect exciton reveals significantly large binding energy, large radius and small effective mass, which are beneficial to the high‐temperature excitonic condensation. In particular, the high‐temperature excitonic BEC can be speculated at 289.7 K. In addition, the exciton current in BKT phase can travel in material without resistance at 72.4 K, indicative of non‐dissipative superfluidity transport. In particular, the results are predominantly derived from ground‐state approaches widely used in current stage, while further exploration on the excitonic condensation based on dynamics methods remains an open question.^[^ ^90^ ^]^

## 4 Experimental Section

In this work, the first‐principles calculations were performed with Perdew–Burke–Ernzerhof (PBE) functional in the framework of generalized gradient approximation (GGA), as implemented in the Vienna ab initio Simulation Package (VASP).^[^ ^91^, ^92^, ^93^ ^]^ In order to eliminate the spurious interactions between periodic images, a vacuum space larger than 30 Å was added in *z* direction. The cutoff kinetic energy for plane waves was set to 500 eV. Structures were fully relaxed until the force on each atom was less than 0.01 eV Å^−1^, and the convergence tolerance for energy was 10^−5^ eV. A Monkhorst–Pack *k*‐point mesh of 9 × 9 × 1 was used to sample the Brillouin zone for geometry optimization and static electronic structural calculations.^[^ ^94^ ^]^ As the starting data for the subsequent many‐body calculations, the Kohn–Sham wave function and charge density was obtained from the Quantum Espresso (QE) code with a 80 Ry kinetic energy cutoff and a 23 × 23 × 1 *k*‐point grid.^[^ ^95^ ^]^ Based on the many‐body Green's function perturbation theory, which is parameterized by the YAMBO distribution, GW approximation as well as the two‐particle BSE were considered to attain the correct excited‐state properties and optical absorption spectra.^[^ ^45^, ^46^ ^]^ In consideration of SOC, Troullier–Martins norm‐conserving fully relativistic pseudopotential was employed for the plane wave functions.^[^ ^96^ ^]^ In particular, PBE energies were corrected by one‐shot G_0_W_0_ approximation to attain the QP eigenvalues. As for the self‐energy and dynamical dielectric screening, 500 bands were employed. A Coulomb cutoff of the screened potential was used in both G_0_W_0_ and optical absorption calculations. In the BS kernel, nine highest valence bands and nine lowest conduction bands were considered.

## Conflict of Interest

The authors declare no conflict of interest.

## Supporting information

Supporting Information

## Data Availability

The data that support the findings of this study are available in the supplementary material of this article.
