# Supplementary material for: High‐Temperature Excitonic Condensation in 2D Lattice
Source: Adv Sci (Weinh). 2024 Sep 6;11(41):2404436. doi: 10.1002/advs.202404436 (PMC11538676; doi:10.1002/advs.202404436)
Supplement: Supplementary file 1 — Supporting Information [file ADVS-11-2404436-s001.docx]

**Supporting Information**

**High-Temperature Excitonic Condensation in Two-Dimensional Lattice**

Yushuo Xu ^a^, Yuanyuan Wang ^b,^ *, Shiqiang Yu ^a^, Dongyue Sun ^a^, Ying Dai ^a,^ *, Baibiao Huang ^a^, and Wei Wei ^a,^ *

^a^ *School of Physics, State Key Laboratory of Crystal Materials, Shandong University, Jinan 250100, China*

^b^ *Science, Mathematics and Technology Cluster, Singapore University of Technology and Design, 487372, Singapore*

* Corresponding authors: yuanyuan2_wang@sutd.edu.sg (Y. Wang), daiy60@sdu.edu.cn (Y. Dai), weiw@sdu.edu.cn (W. Wei)

1. **Interband Transition Matrix**

On the basis of perturbation theory, the Hamiltonian in a perturbing electric–magnetic (EM) field reads

$$H=\frac{1}{2}\sum_{i} \frac{1}{m}\left( \boldsymbol{p}_{i}+\frac{\left| e \right|\boldsymbol{A}\left( \boldsymbol{r}_{i} \right)}{c} \right)^{2}+\sum_{i} e\phi\left( \boldsymbol{r}_{i} \right)+\sum_{i} V\left( \boldsymbol{r}_{i} \right)=H_{0}+H_{p}$$

where $\boldsymbol{A}\left( \boldsymbol{r}_{i} \right)$ and $\phi\left( \boldsymbol{r}_{i} \right)$ stand for the vector potential and scalar potential of EM field, respectively, and $H_{0}$ is the unperturbed Hamiltonian, thus

$$H_{p}=\frac{\left| e \right|}{2mc}\sum_{i} \left( \boldsymbol{p}_{i}\cdot\boldsymbol{A}\left( \boldsymbol{r}_{i} \right)+\boldsymbol{A}\left( \boldsymbol{r}_{i} \right)\cdot\boldsymbol{p}_{i} \right)+\frac{1}{2c^{2}}\sum_{i} \frac{e^{2}}{m}A^{2}\left( \boldsymbol{r}_{i} \right)+\sum_{i} e\phi\left( \boldsymbol{r}_{i} \right)$$

If we select the Coulomb gauge, then we have

$$\nabla\cdot\boldsymbol{A=}0$$

$$\phi=0$$

for EM field.

In consideration of a small $\boldsymbol{A}\left( \boldsymbol{r}_{i} \right)$ and neglecting high-order terms, the *i*-th electron perturbed Hamiltonian can be simplified as

$$H_{p}^{i}\approx-\frac{e}{mc}\boldsymbol{A}\left( \boldsymbol{r}_{i} \right)\cdot\boldsymbol{p}_{i}=i\frac{e\hbar}{mc}\boldsymbol{A}\left( \boldsymbol{r}_{i} \right)\cdot\nabla$$

Vector potential $\boldsymbol{A}\left( \boldsymbol{r}_{i} \right)$ in EM wave can be expressed as

$$\boldsymbol{A}\left( \boldsymbol{r}_{i} \right)=\frac{\boldsymbol{A}_{0}}{2}\left[ e^{i\left( \boldsymbol{q}\cdot\boldsymbol{r}-\omega t \right)}-e^{-i\left( \boldsymbol{q}\cdot\boldsymbol{r}-\omega t \right)} \right]$$

According to the Maxwell’s equations for the EM wave, we have

$$\boldsymbol{E}=-\frac{1}{c}\frac{\partial\boldsymbol{A}}{\partial t}$$

That is,

$$i\omega\boldsymbol{A=}c\boldsymbol{E}$$

Thus,

$$H_{p}^{i}=\frac{e\hbar}{m\omega}e^{i\left( \boldsymbol{q}\cdot\boldsymbol{r}-\omega t \right)}\frac{\boldsymbol{E}_{0}}{2}\cdot\boldsymbol{\nabla}+h.c.$$

Optically, the imaginary part of the dielectric function ($\epsilon=\epsilon_{1}+i\epsilon_{2}$) depends on the transition rate $\epsilon_{2}\left( \omega\right)\propto\omega_{n,\boldsymbol{k};n^{'}\boldsymbol{k}^{'}}$

$$\omega_{n,\boldsymbol{k};n^{'}\boldsymbol{k}^{'}}=\frac{2\pi}{\hbar}\left| \left. \left\langle n^{'}\boldsymbol{,k}^{'} \right. \right|H_{p}\left| \left. n,\boldsymbol{k} \right\rangle\right. \right|^{2}\rho\left( \omega,E \right)$$

$$=\frac{\pi\hbar e^{2}E_{0}^{2}}{2m^{2}\omega^{2}}\left| \vec{\boldsymbol{e}}\cdot\boldsymbol{M}_{nn^{'}}\left( \boldsymbol{k},\boldsymbol{k}^{'} \right) \right|^{2} \rho\left( \omega,E \right)$$

where $\rho\left( \omega,E \right)$ represents the density of states, and the transition matrix element reads^[1]^

$$\boldsymbol{M}_{nn^{'}}\left( \boldsymbol{k},\boldsymbol{k}^{'} \right)=\left. \left\langle n^{'},\boldsymbol{k}^{'} \right. \right|e^{i\boldsymbol{q}\cdot\boldsymbol{r}}\boldsymbol{\nabla}\left| \left. n,\boldsymbol{k} \right\rangle\right.$$

$$=\int\mathcal{u}_{n^{'}\boldsymbol{k}^{'}}^{*}e^{-i\boldsymbol{k}^{'}\cdot\boldsymbol{r}}e^{i\boldsymbol{q}\cdot\boldsymbol{r}}\boldsymbol{\nabla}\left( \mathcal{u}_{n\boldsymbol{k}}e^{i\boldsymbol{k}\cdot\boldsymbol{r}} \right)d^{3}r$$

$$=\int\mathcal{u}_{n^{'}\boldsymbol{k}^{'}}^{*}e^{-i\left( \boldsymbol{k}^{'}-\boldsymbol{q}-\boldsymbol{k} \right)\cdot\boldsymbol{r}}\left( \boldsymbol{\nabla+}i\mathbf{k} \right)\mathcal{u}_{n\boldsymbol{k}}d^{3}r$$

Accordingly, transition matrix element has finite value only when

$$\boldsymbol{k}^{'}-\boldsymbol{q}-\boldsymbol{k=}0 or\boldsymbol{G}$$

Generally, the photoexcitation undergoes the direction transitions, that is, $\boldsymbol{q\sim}0$ and $\boldsymbol{k}^{'}\sim\boldsymbol{k}$. Therefore, transition matrix element indicates simplified form

$$\boldsymbol{M}_{nn^{'}\boldsymbol{k}}=\left. \left\langle n^{'}\boldsymbol{,k} \right. \right|\boldsymbol{\nabla}\left| \left. n,\boldsymbol{k} \right\rangle\right.$$

Considering the transition in the light cone, transition matrix element can be rewritten as

$$\boldsymbol{M}_{nn^{'}\boldsymbol{k}}=\left. \left\langle n^{'}\boldsymbol{,k+q} \right. \right|e^{i\boldsymbol{q}\cdot\boldsymbol{r}}\boldsymbol{\nabla}\left| \left. n,\boldsymbol{k} \right\rangle\right.$$

here, $\boldsymbol{q=}\frac{\omega}{c}\boldsymbol{\sim}0$.

1. **Electric Dipole Approximation**

It is well known that the electric component dominates in the electric-magnetic interaction between electron system and light field. Without loss of generality, electric field intensity in EM field propagating along *z* axis can be expressed as

$$\mathcal{E}=\frac{\mathcal{E}_{x}\boldsymbol{i}+\mathcal{E}_{y}\boldsymbol{j}}{2}\left( e^{i\left( \frac{2\pi z}{\lambda}-\omega t \right)}+e^{-i\left( \frac{2\pi z}{\lambda}-\omega t \right)} \right)$$

$$=\left( \mathcal{E}_{x}\boldsymbol{i}+\mathcal{E}_{y}\boldsymbol{j} \right)\cos\left( \frac{2\pi z}{\lambda}-\omega t \right)$$

Thus, the *i*-th electron perturbed Hamiltonian reads

$$H_{p}^{i}=-\boldsymbol{\mu}\cdot\mathcal{E}$$

where $\boldsymbol{\mu}$ symbolizes the electric dipole.

The Taylor expansion of cosine function with respect to *z* variable has

$$\cos\left( \frac{2\pi z}{\lambda}-\omega t \right)=\cos\omega t-\frac{2\pi z}{\lambda}\sin\omega t+\cdots$$

Actually, the value of *z* variable is the same order of magnitude as the atom radius, while light wave length is four orders of magnitude larger than atom radius. Therefore, we only take the first term in Taylor expansion, that is, the electric dipole approximation.

The direct transition rate $\omega_{n,n^{'}\boldsymbol{,k}}$ follows

$$\omega_{n,n^{'}\boldsymbol{,k}}=\left( \frac{\pi{\mathcal{E}_{x}}^{2}}{2\hbar}\left| \hat{\mu}_{n,n^{'}\boldsymbol{,k}}^{x} \right|^{2}+\frac{\pi{\mathcal{E}_{y}}^{2}}{2\hbar}\left| \hat{\mu}_{n,n^{'}\boldsymbol{,k}}^{y} \right|^{2} \right)\rho\left( \omega,E \right)$$

In this equation, $\hat{\mu}_{n,n^{'}\boldsymbol{,k}}^{i}$ is the transition dipole moment, which has a concise form as^[2]^

$$\hat{\mu}_{n,n^{'}}=\left. \left\langle\varphi_{n^{'}} \right. \right|\hat{\boldsymbol{\mu}}\left| \left. \varphi_{n} \right\rangle\right.$$

1. **Relation between Transition Dipole Moment and Transition Matrix Element**

We first summarize the different forms for the direction transition in two situations. The transition matrix element reads

$$\boldsymbol{M}_{nn^{'}\boldsymbol{k}}=\left. \left\langle n^{'}\boldsymbol{,k} \right. \right|\boldsymbol{\nabla}\left| \left. n,\boldsymbol{k} \right\rangle\right.$$

$$=\frac{i}{\hbar}\left. \left\langle n^{'}\boldsymbol{,k} \right. \right|\hat{\boldsymbol{p}}\left| \left. n,\boldsymbol{k} \right\rangle\right.$$

and the transition dipole moment follows

$$\hat{\mu}_{n,n^{'}\boldsymbol{,k}}=\left. \left\langle n^{'}\boldsymbol{,k} \right. \right|\hat{\boldsymbol{\mu}}\left| \left. n,\boldsymbol{k} \right\rangle\right.$$

Rigorously, the Hamiltonian in this system reads

$$H=H_{0}+i\frac{e\hbar}{mc}\boldsymbol{A}\left( \boldsymbol{r}_{i} \right)\cdot\nabla+\frac{e^{2}}{2mc^{2}}A^{2}\left( \boldsymbol{r}_{i} \right)$$

Then

$$\left[ \hat{\boldsymbol{\mu}},H \right]=-i\hbar e\frac{\hat{\boldsymbol{p}}}{m}+\frac{e^{2}}{mc}\boldsymbol{A}\cdot{\vec{\boldsymbol{e}}}_{\boldsymbol{p}}$$

Therefore, we have

$$\left( \varepsilon_{n}-\varepsilon_{n^{'}} \right)\left. \left\langle n^{'}\boldsymbol{,k} \right. \right|\hat{\boldsymbol{\mu}}\left| \left. n,\boldsymbol{k} \right\rangle\right.=-\frac{i\hbar e}{m}\left. \left\langle n^{'}\boldsymbol{,k} \right. \right|\hat{\boldsymbol{p}}\left| \left. n,\boldsymbol{k} \right\rangle\right.\mathcal{+O}\left( {n,n}^{'}\boldsymbol{,k} \right)$$

with $\mathcal{O}\left( {n,n}^{'}\boldsymbol{,k} \right)=\frac{e^{2}}{mc}\left. \left\langle n^{'}\boldsymbol{,k} \right. \right|\hat{\boldsymbol{A}}\boldsymbol{\cdot}{\vec{\boldsymbol{e}}}_{\boldsymbol{p}}\left| \left. n,\boldsymbol{k} \right\rangle\right.$ being residual term, which is omitted.

Therefore, we have the relation

$$\left. \left\langle n^{'}\boldsymbol{,k} \right. \right|\boldsymbol{\nabla}\left| \left. n,\boldsymbol{k} \right\rangle\right.=\frac{m\left( \varepsilon_{n^{'}}-\varepsilon_{n} \right)}{e\hbar^{2}}\left. \left\langle n^{'}\boldsymbol{,k} \right. \right|\hat{\boldsymbol{\mu}}\left| \left. n,\boldsymbol{k} \right\rangle\right.$$

$$=\gamma\left. \left\langle n^{'}\boldsymbol{,k} \right. \right|\hat{\boldsymbol{\mu}}\left| \left. n,\boldsymbol{k} \right\rangle\right.$$

It can be deduced that two forms share the similar physical mechanism, in which the difference stems from an amplification coefficient.

1. **Optical Selective Rules for Excitons with Different Angular Quantum Number**

Theoretically, the oscillator strength can be expressed as^[3,4]^

$$I_{e}^{S}=\frac{2\hbar^{2}\left| \sum_{\boldsymbol{k}} A_{\boldsymbol{k}}^{S}\boldsymbol{e}\cdot\boldsymbol{M}_{nn^{'}k} \right|^{2}}{E_{S}}$$

The exciton envelope function satisfies the BSE equation^[5]^

$$\left( {\varepsilon_{n^{'}}-\varepsilon}_{n} \right)A_{k}^{S}+\sum_{\boldsymbol{q}} A_{\boldsymbol{q}}^{S}\left. \left\langle n,n^{'}\boldsymbol{,k} \right. \right|\hat{K}^{eh}\left| \left. n,n^{'}\boldsymbol{,k+q} \right\rangle\right.=E_{S}A_{k}^{S}$$

It can be deduced that exciton envelope function and transition matrix element depend on the *k* points. Thus, the oscillator strength can be rewritten in a concise form as

$$I_{e}^{S}=\frac{2\hbar^{2}\left| \sum_{\boldsymbol{k}} f\left( \left| \boldsymbol{k} \right| \right)e^{i\left( m+l \right)\theta_{k}} \right|^{2}}{E_{S}}$$

where $f\left( \left| \boldsymbol{k} \right| \right)$ is the radial part, *m* and *l* symbolize the angular quantum numbers and winding numbers of transition matrix element under a specific polarized light, respectively. In particular, $I_{e}^{S}$ has finite value only when $m=-l \left( \mathrm{mod} n \right)$.

1. **Deviation of Two-Dimensional Hydrogen-Atom Model**

Considering a system containing two interacting particles, the interaction between two particles is

$$V\left( \boldsymbol{r}_{1},\boldsymbol{r}_{2} \right)=V\left( \boldsymbol{r}_{1}-\boldsymbol{r}_{2} \right)$$

then, the Hamiltonian of this system reads

$$i\hbar\frac{\partial}{\partial t}\Psi\left( \boldsymbol{r}_{1},\boldsymbol{r}_{2},t \right)=\left[ -\frac{\hbar^{2}}{2m_{1}}\nabla_{1}^{2}-\frac{\hbar^{2}}{2m_{2}}\nabla_{2}^{2}+V\left( \boldsymbol{r}_{1},\boldsymbol{r}_{2} \right) \right]\Psi\left( \boldsymbol{r}_{1},\boldsymbol{r}_{2},t \right)$$

By introducing center-of-mass coordinate, the Hamiltonian turns out to be

$$i\hbar\frac{\partial}{\partial t}\Psi\left( \boldsymbol{R},\boldsymbol{r},t \right)=\left[ -\frac{\hbar^{2}}{2M}\nabla_{R}^{2}-\frac{\hbar^{2}}{2\mu}\nabla_{r}^{2}+V\left( \boldsymbol{r} \right) \right]\Psi\left( \boldsymbol{R},\boldsymbol{r},t \right)$$

where $M=m_{1}+m_{2}$ and $\mu=\frac{m_{1}m_{2}}{m_{1}+m_{2}}$.

In accordance to the separation of variables

$$\Psi\left( \boldsymbol{r}_{1},\boldsymbol{r}_{2},t \right)=\phi\left( \boldsymbol{R} \right)\varphi\left( \boldsymbol{r} \right)\chi\left( t \right)$$

Thus,

$$\chi\left( t \right)=e^{-i{E_{t}}/\hbar}=e^{-i\left( E_{c}+E \right)/\hbar}$$

$$-\frac{\hbar^{2}}{2M}\nabla_{R}^{2}\phi\left( \boldsymbol{R} \right)=E_{c}\phi\left( \boldsymbol{R} \right)$$

$$\left( -\frac{\hbar^{2}}{2\mu}\nabla_{r}^{2}+V\left( \boldsymbol{r} \right) \right)\varphi\left( \boldsymbol{r} \right)=E\varphi\left( \boldsymbol{r} \right)$$

For the interaction between electron and hole, $V\left( \boldsymbol{r} \right)$ is determined by the attractive Coulomb potential, that is,

$$V\left( \boldsymbol{r} \right)=-\frac{e^{2}}{r}$$

In polar coordinates, the Schrödinger equation is transformed to

$$\left[ -\frac{\hbar^{2}}{2\mu}\left( \frac{\partial^{2}}{\partial r^{2}}+\frac{1}{r}\frac{\partial}{\partial r}+\frac{1}{r^{2}}\frac{\partial^{2}}{\partial\theta^{2}} \right)-\frac{e^{2}}{r} \right]\varphi\left( \boldsymbol{r,}\theta\right)=E\varphi\left( \boldsymbol{r,}\theta\right)$$

The analytic eigenvalues of the above equation in bound discrete states are derived to

$$E_{n}=-\frac{1}{2\left( n-\frac{1}{2} \right)^{2}}\frac{\mu e^{4}}{\hbar^{2}},n=1,2,3,\ldots$$

and the normalized radial eigenfunctions are^[6]^

$$R_{10}=\xi_{1}e^{-\xi_{1}r/2}$$

$$R_{20}=\left( {\xi_{2}}/{3^{1/2}} \right)\left( 1-\xi_{2}r \right)e^{-\xi_{2}r/2}$$

$$R_{21}=\left( {\xi_{2}^{2}}/{6^{1/2}} \right)re^{-\xi_{2}r/2}$$

$$\cdots$$

Here, $\xi_{n}=\frac{2}{n-\frac{1}{2}}\frac{\mu e^{2}}{\hbar^{2}}$.

Accordingly, the average values of the average radius and root mean square radius read

$$\left( \bar{r} \right)_{nl}=\frac{1}{2}\left[ 3n\left( n-1 \right)-\left| l \right|^{2}+1 \right]a_{0}$$

$$\left( \bar{r^{2}} \right)_{nl}=\frac{1}{8}\left( 2n-1 \right)\left[ n\left( 10n^{2}-15n+11 \right)-3\left| l \right|^{2}\left( 2n-1 \right)-3 \right]a_{0}$$

with $a_{0}=\frac{\hbar^{2}}{\mu e^{2}}$ being the Bohr radius.^[7]^

In this sense, we can conclude the ratios for the first three eigenvalues and between average radius and root mean square radius

$$E_{b_{1s}}:E_{b_{2s}}:E_{b_{3s}}=30:5:3$$

$$r_{\mathrm{RMS}}:r_{\mathrm{ex}}=\sqrt{2/3}$$

Indeed, the eigenvalues of the first three excitons do not follow the above ratio. This can be attributed to the dielectric environment. The dielectric function in two-dimensional (2D) Bi_2_S_2_Te can be expressed as

$$\epsilon\left( \omega\right)=1+\left( 4\pi\alpha\left( \omega\right) \right)/d$$

In this expression, $\alpha\left( \omega\right)$ represents the 2D polarization function,^[8,9]^ which is obtained from the Yambo code including excitonic effect and local-field effects. The Coulomb potential is rewritten as $V\left( \boldsymbol{r} \right)=-\frac{e^{2}}{\epsilon r}$. Upon the correction of the dielectric function, the first three eigenvalues substantially follow the 2D hydrogen-like stationary state energy.

1. **Saha Temperature of Exciton in Two Dimensions**

Assuming a 2D excitonic system with *N*_x0_ excitons in a volume of *V*_2D_. Upon thermal dissociation, there are *N*_ex_ excitons and *N*_e_ (*N*_h_) electrons (holes), satisfying

$$N_{x0}=N_{\mathrm{ex}}+N_{e}$$

Statistical physics implies that the probability of the exciton with a binding energy $E_{b}$ reads

$$P_{\mathrm{ex}}=Ae^{\frac{E_{b}}{k_{B}T}}$$

here coefficient $A$ can be deduced from the thermodynamic condition as

$$A=\left( \exp\left( \frac{E_{b}}{k_{B}T} \right)+\frac{1}{\lambda_{B}^{2}n_{e}} \right)^{-1}$$

where $\lambda_{B}=\sqrt{\frac{h^{2}}{2\pi M_{ex}k_{B}T}}$ is the De Broglie wavelength of excitons for thermal motion.

Consequently, the probabilities of the exciton and electron have

$$P_{\mathrm{ex}}=\frac{\exp\left( {E_{b}}/{k_{B}T} \right)}{\exp\left( {E_{b}}/{k_{B}T} \right)+1/{\lambda_{B}^{2}n_{e}}}$$

$$P_{e}=P_{h}=1-P_{\mathrm{ex}}=\frac{1/{\lambda_{B}^{2}n_{e}}}{\exp\left( {E_{b}}/{k_{B}T} \right)+1/{\lambda_{B}^{2}n_{e}}}$$

Then, we have

$$\frac{n_{e}n_{h}}{n_{\mathrm{ex}}}=\frac{1}{\lambda_{B}^{2}}e^{-\frac{E_{b}}{k_{B}T}}$$

Furthermore, considering the degree of freedom of particles, one has

$$\frac{\alpha^{2}}{1-\alpha}=\frac{g_{h}g_{e}}{g_{\mathrm{ex}}}\frac{e^{-\frac{E_{b}}{k_{B}T}}}{\lambda_{B}^{2}n_{\mathrm{ex}}}$$

Here, $\alpha$ represents the ionization coefficient. This is the Saha equation of exciton in two dimensions.^[10]^

In particular, if we adopt $\alpha=0.414$, the Saha equation has

$$k_{B}T_{s}=\left( \frac{\pi\hbar^{2}}{M_{\mathrm{ex}}}n_{\mathrm{ex}} \right)e^{\frac{E_{b}}{k_{B}T_{s}}}$$

In the normal situation, we have

$$k_{B}T_{s}\sim\left( \frac{\pi\hbar^{2}}{M_{\mathrm{ex}}}n_{\mathrm{ex}} \right)e^{\frac{E_{b}}{k_{B}T_{s}}}$$

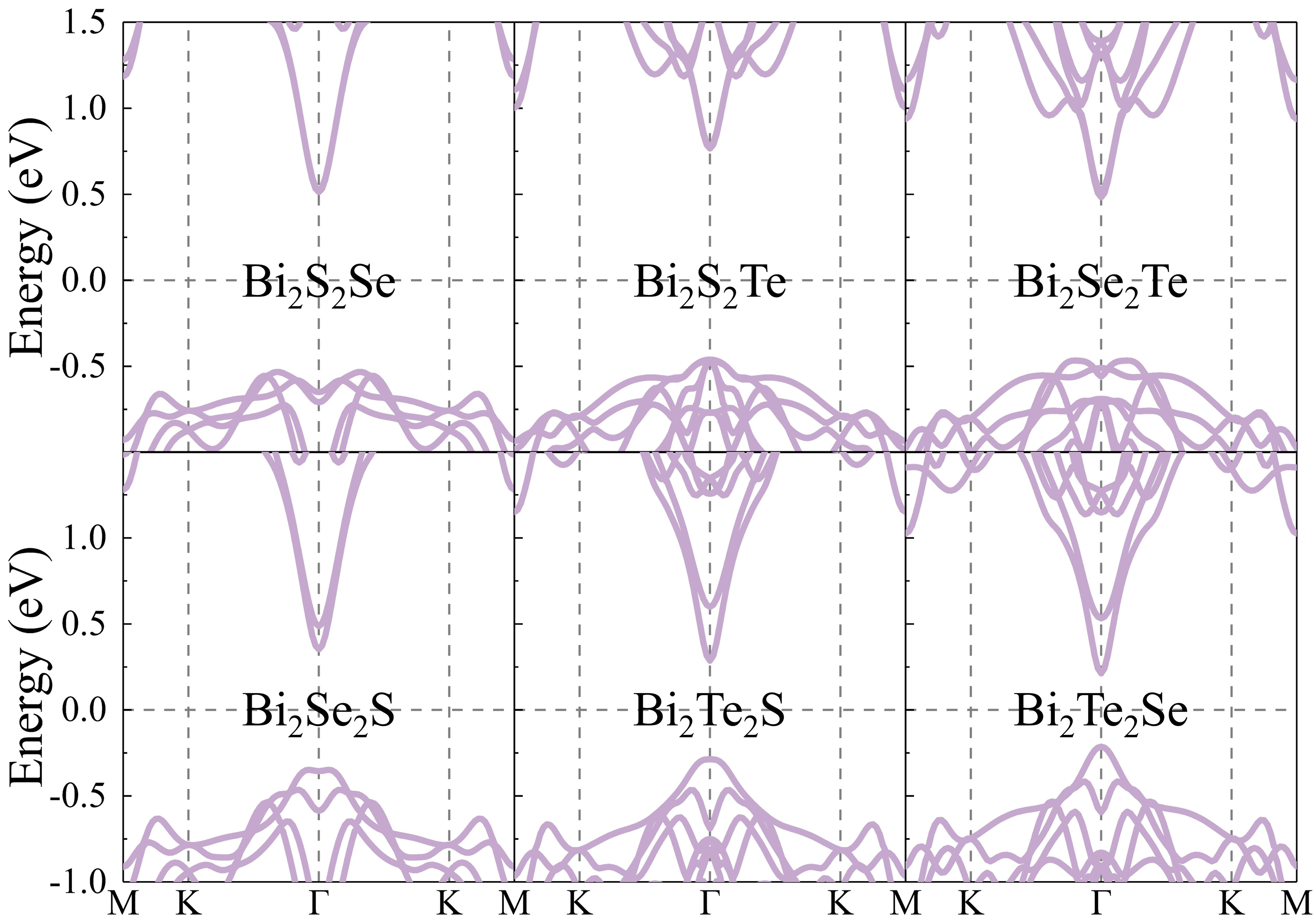


**Figure S1.** Band structures for Bi_2_X_2_Y (X ≠ Y = S, Se, Te) in bulk phase at PBE level of theory without the consideration of SOC.


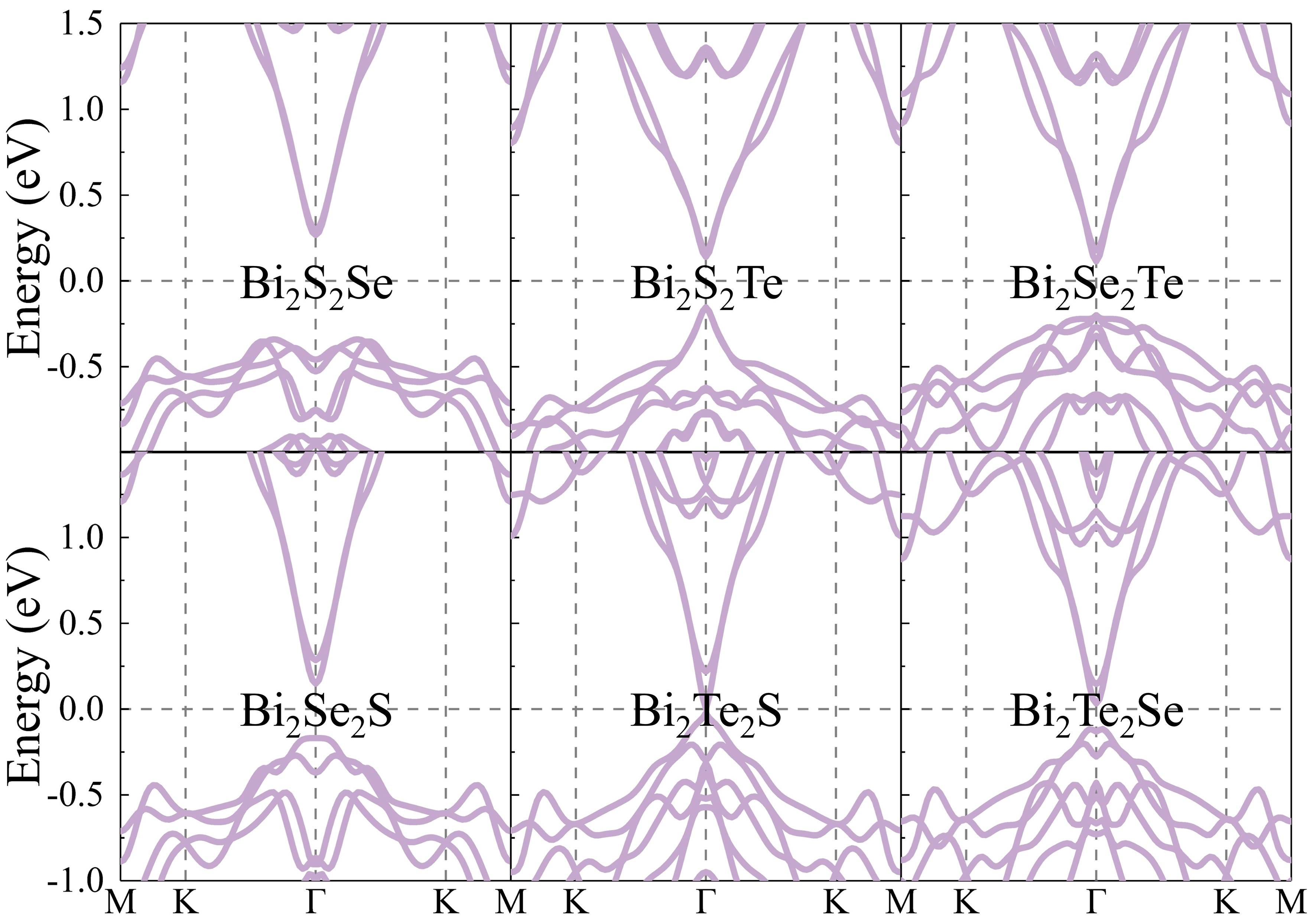


**Figure S2.** Band structures f Bi_2_X_2_Y (X ≠ Y = S, Se, Te) in bulk phase at PBE level of theory with the consideration of SOC.


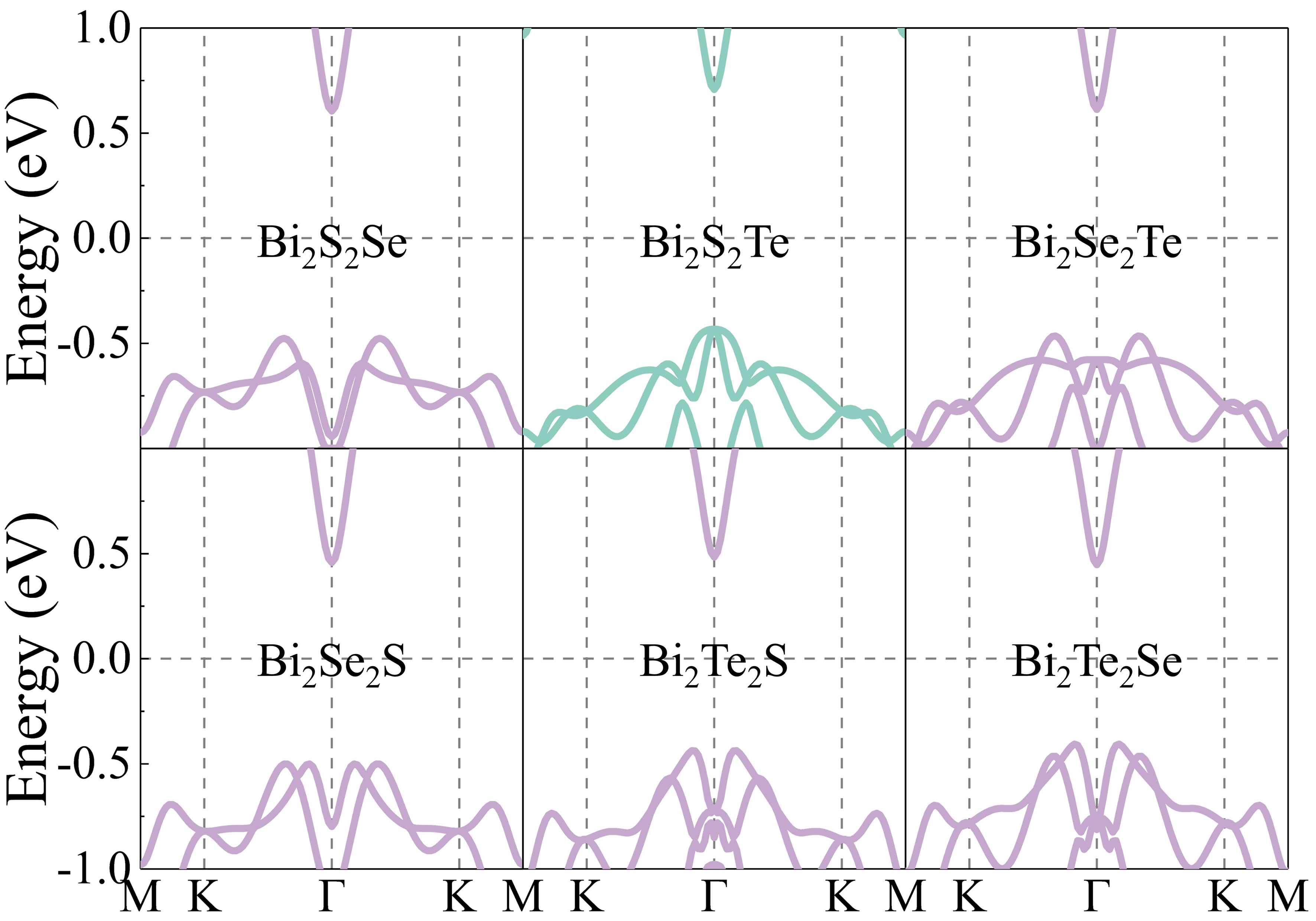


**Figure S3.** Band structures for Bi_2_X_2_Y (X ≠ Y = S, Se, Te) monolayer at PBE level of theory without the consideration of SOC.


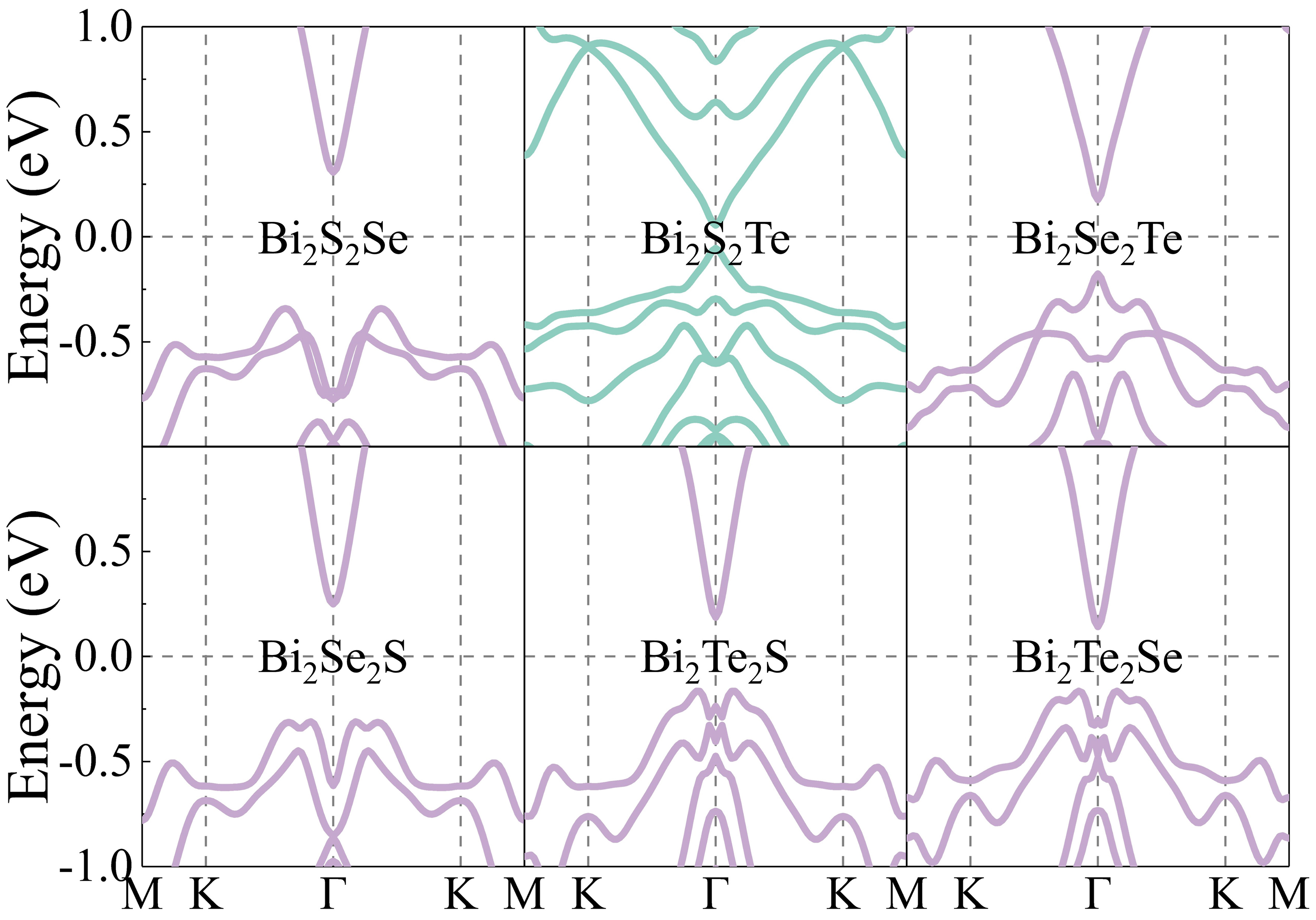


**Figure S4.** Band structures for Bi_2_X_2_Y (X ≠ Y = S, Se, Te) monolayer at PBE level of theory with the consideration of SOC.


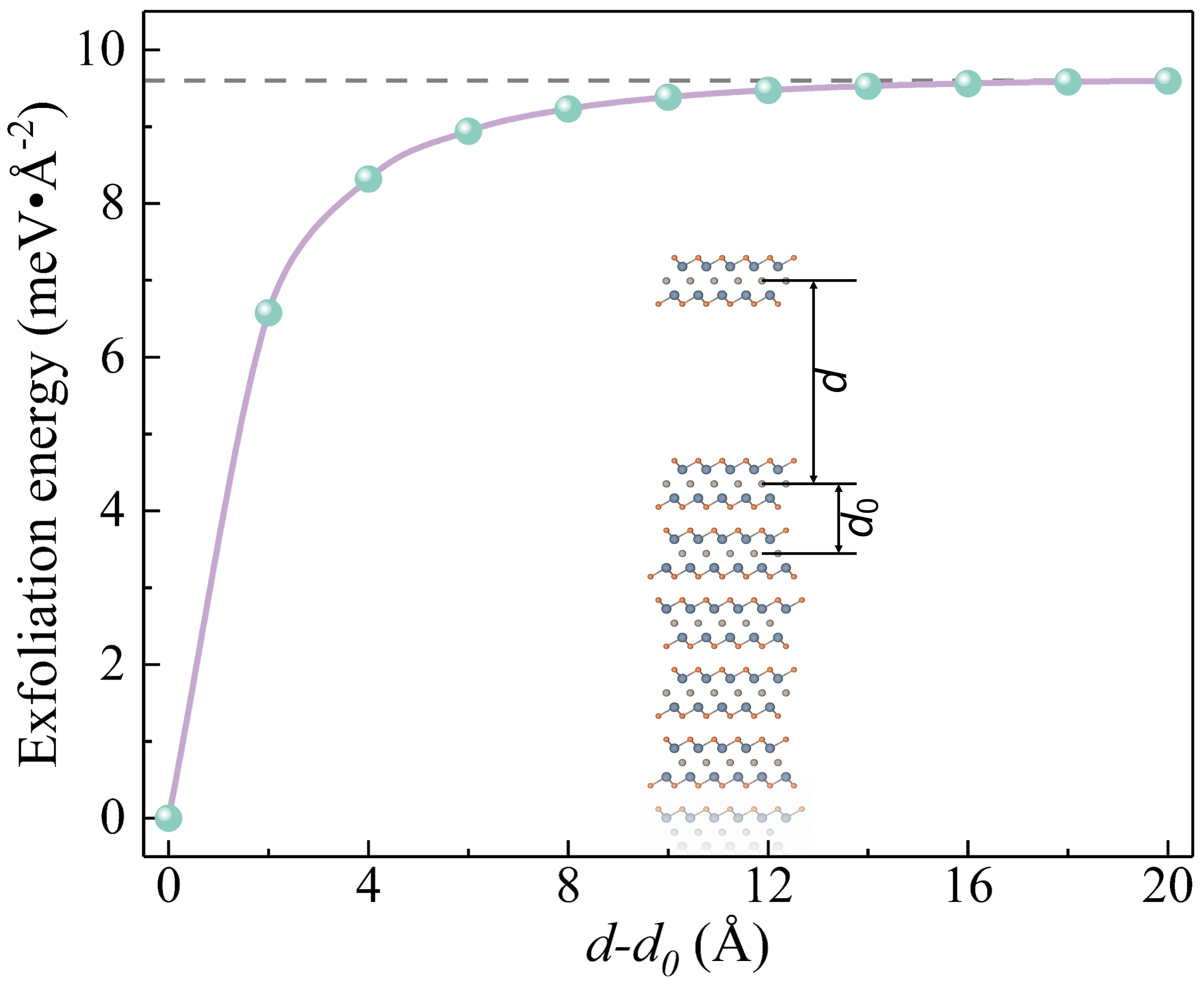


**Figure S5.** Exfoliation energy for Bi_2_S_2_Te monolayer. Inset shows the sketch of exfoliation with *d* and *d*_0_ being interlayer distance and original van der Waals (vdW) distance, respectively.


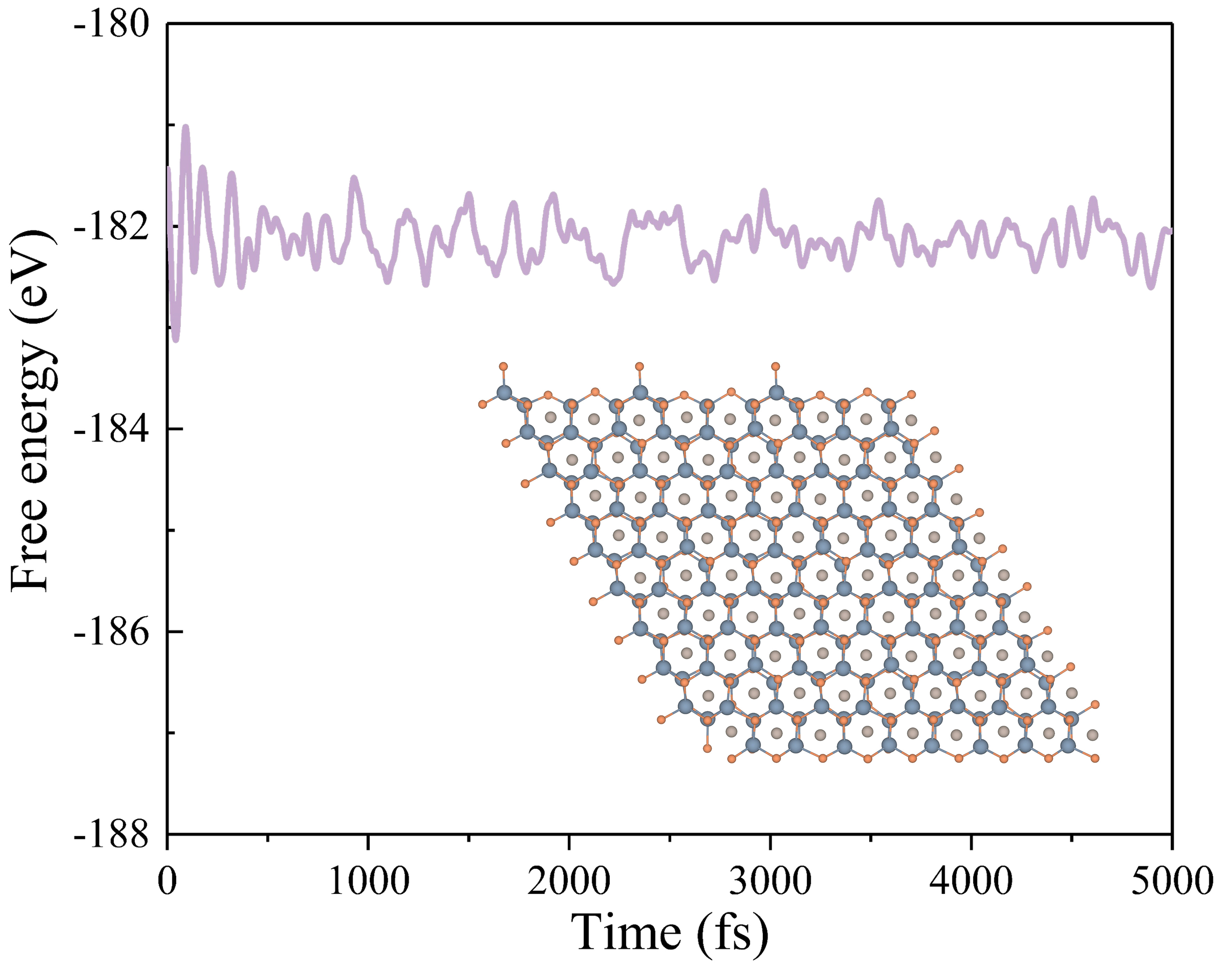


**Figure S6.** Free energy variation of Bi_2_S_2_Te monolayer at 300 K from the *ab initio* molecular dynamics (AIMD) simulations. Inset shows the final structure after 5 ps.


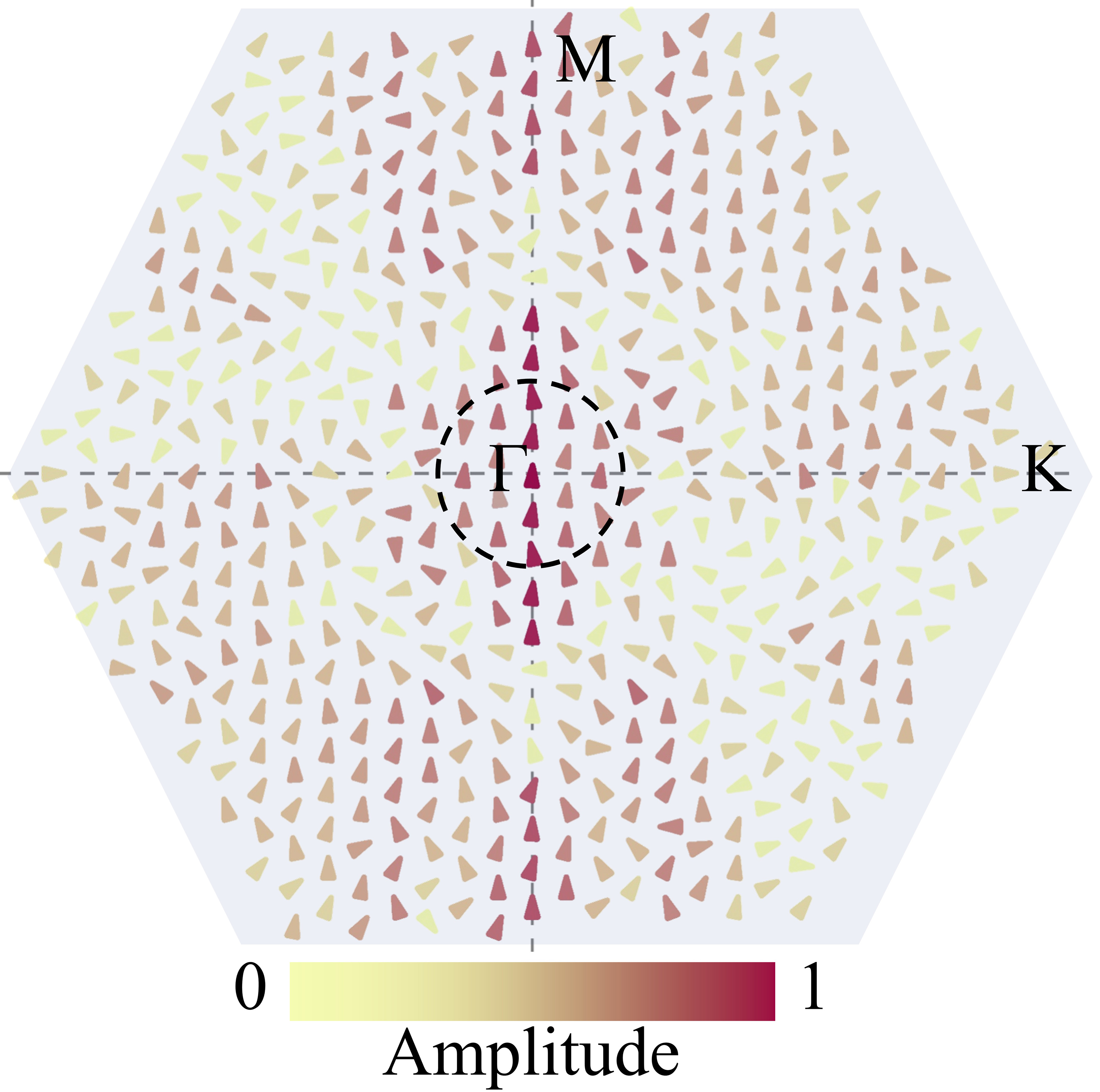


**Figure S7.** Phase and amplitude of the interband optical matrix element over the entire Brillion zone. The cylindrical angular quantum number of the lowest-energy 1*s* exciton state is set to zero.

**References**

1. M. L. Cohen, S. G. Louie, *Fundamentals of Condensed Matter Physics.* Cambridge University Press, Cambridge **2016**.
2. J. Luo, X. Wang, S. Li, J. Liu, Y. Guo, G. Niu, L. Yao, Y. Fu, L. Gao, Q. Dong, C. Zhao, M. Leng, F. Ma, W. Liang, L. Wang, S. Jin, J. Han, L. Zhang, J. Etheridge, J. Wang, Y. Yan, E. H. Sargent, J. Tang, *Nature* **2018**, *563*, 541.
3. R. J. Elliott, *Phys. Rev.* **1957**, *108*, 1384.
4. J. Deslippe, G. Samsonidze, D. A. Strubbe, M. Jain, M. L. Cohen, S. G. Louie, *Comput. Phys. Commun.* **2012**, *183*, 1269.
5. M. Rohlfing, S. G. Louie, *Phys. Rev. B* **2000**, *62*, 4927.
6. X. L. Yang, S. H. Guo, F. T. Chan, K. W. Wong, W. Y. Ching, *Phys. Rev. A* **1991**, *43*, 1186.
7. L. D. Landau, E. M. Lifshitz, *Quantum Mechanics*, Pergamon, New York **1981**.
8. D. Wang, N. Luo, W. Duan, X. Zou, *J. Phys. Chem. Lett.* **2021**, *12*, 5479.
9. A. Molina-Sánchez, G. Catarina, D. Sangalli, J. Fernánadez-Rossier, *J. Mater. Chem. C* **2020**, *26*, 3441.
10. M. N. Saha, *Phil. Mag.* **1920**, *128*, 997.
